# A commentary on ‘The safety and efficacy between remimazolam and propofol in intravenous anesthesia of endoscopy operation: a systematic review and meta-analysis’

**DOI:** 10.1097/JS9.0000000000001439

**Published:** 2024-04-04

**Authors:** Hongxu Ren, Zhaojie Lv, Zijun Liu, Haibo Wang

**Affiliations:** a Department of Operating Room, Yantai Yuhuangding Hospital; b Department of Anesthesiology, Yantai Yuhuangding Hospital; c Department of Gastrointestinal Surgery, Yantai Yuhuangding Hospital, Yantai, Shandong, People’s Republic of China

*Dear Editor,*

It was with great interest that we read the article published in the *International Journal of Surgery*. Zhao *et al*.^1^ conducted a meta-analysis of patients with American Society of Anesthesiologists (ASA-PS) scores of grade Ⅰ or Ⅱ who underwent endoscopic surgery in outpatient operating rooms. Studies imply that for endoscopic procedures, remimazolam can be a more secure shock alternative than propofol.

Endoscopic treatments are frequently carried out on older people and are highly beneficial in the early detection and screening of gastrointestinal disorders^2^. The need for comfortable medical care has led to the widespread use of painless endoscopic treatments. The most often given drugs for sedation during endoscopic procedures globally are midazolam, etomidate, and propofol. Merely consuming etomidate may result in tremors and rigidity in the muscles, as well as exacerbate postoperative nausea and vomiting (PONV). These side effects could potentially affect the surgery and lower patient and endoscopist satisfaction. Nurses in the ward are therefore required to care for individuals who have choked or experienced other problems. Injection pain, loss of protective reflexes leading to aspiration pneumonia, lack of analgesic effect and specific antagonists, metabolic acidosis, risk of developing propofol infusion syndrome and bacterial contamination, narrow therapeutic index (raising the risk of hypoxia), and depression of the cardiovascular and respiratory systems are the drawbacks of propofol. Anesthesia nursing and operating room nursing during anesthetic will be especially critical, which means finding complications in time and giving treatment. Due to its elimination half-life of 1.8–6.6 h, midazolam has some drawbacks as well, including prolonged post-procedural drowsiness and longer hospital stays, particularly in patients with renal or hepatic failure^3^. That means more nurses in the ward were required to monitor the occurrence of postoperative complications. Anaesthesiologists therefore constantly look for sedatives that can be used for endoscopic procedures on elderly patients that have good sedative effects and very few side effects.

The ultrashort-acting benzodiazepine remimazolam was created recently. It has a strong affinity for gamma-aminobutyric acid receptors and raises the frequency at which chloride channels in the cerebral cortex, limbic system, midbrain, brainstem, and spinal cord open^4,5^. Remimazolam’s and Remimazolam tosilate’s pharmacokinetics and pharmacodynamics are comparable. Remimazolam has a quicker start to action and a shorter half-life than midazolam. Remimazolam is safe and effective for use in general anesthesia and procedural sedation (e.g. gastrointestinal endoscopy and bronchoscopy), according to relevant research at various stages of clinical trials.

This investigation looked at the side effects and comparative effectiveness of propofol and remimazolam during endoscopic procedures. This novel approach provides insightful guidance and inspiration for the further development of this field of study. However, there were a few limitations. First, the assumption made when conducting this meta-analysis was that all of the patients who were a part of the studies had comparable baseline characteristics. High clinical heterogeneity may have resulted from inconsistent baseline features. Second, as the majority of the studies were conducted in China, there may be notable differences in the outcomes between various demographic groups.

In order to get more precise results, more robust clinical trials should be carried out in the future. Further trials in obese populations, intensive care units, and vulnerable groups (such as those with respiratory diseases) may be possible. To improve outcomes, more trials for each of the ASA classifications stated above ought to be conducted. Remimazolam’s overall procedural success is remarkable. However, but before this medication is widely used and accepted, some aspects – like its safety profile and cost-effectiveness – need to be carefully examined.

## Ethical approval

Not applicable.

## Consent

Not applicable.

## Author contribution

H.R. and Z.L.: study concept or design, data collection, data analysis or interpretation, and writing the paper; Z.L.: data collection; H.W.: study concept or design, writing, and revising the paper.

## Conflicts of interest disclosure

The authors declare no conflicts of interest.

## Research registration unique identifying number (UIN)

Not applicable.

## Guarantor

Haibo Wang.

## Data availability statement

Not applicable.

## Provenance and peer review

Not applicable.
